# Embodied Predictions, Agency, and Psychosis

**DOI:** 10.3389/fdata.2020.00027

**Published:** 2020-08-14

**Authors:** Pantelis Leptourgos, Philip R. Corlett

**Affiliations:** Department of Psychiatry, Connecticut Mental Health Center, Yale University, New Haven, CT, United States

**Keywords:** predictive processing, delusions, hallucinations, corollary discharge, psychosis, embodiment, agency

## Abstract

Psychotic symptoms, i.e., hallucinations and delusions, involve gross departures from conscious apprehension of consensual reality; respectively, perceiving and believing things that, according to same culture peers, do not obtain. In schizophrenia, those experiences are often related to abnormal sense of control over one's own actions, often expressed as a distorted sense of agency (i.e., passivity symptoms). Cognitive and computational neuroscience have furnished an account of these experiences and beliefs in terms of the brain's generative model of the world, which underwrites inferences to the best explanation of current and future states, in order to behave adaptively. Inference then involves a reliability-based trade off of predictions and prediction errors, and psychotic symptoms may arise as departures from this inference process, either an over- or under-weighting of priors relative to prediction errors. Surprisingly, there is empirical evidence in favor of both positions. Relatedly, there is evidence for both an enhanced and a diminished sense of agency in schizophrenia. How can this be? We argue that there is more than one generative model in the brain, and that ego- and allo-centric models operate in tandem. In brief, ego-centric models implement corollary discharge signals that cancel out the effects of self-generated actions while allo-centric models compare several hypothesis regarding the causes of sensory inputs (including the self among the potential causes). The two parallel hierarchies give rise to different levels of agency, with ego-centric models subserving “feelings of agency” and allo-centric predictions giving rise to “judgements of agency.” Those two components are weighted according to their reliability and combined, generating a higher-level “sense of agency.” We suggest that in schizophrenia a failure of corollary discharges to suppress self-generated inputs results in the absence of a “feeling of agency” and in a compensatory enhancement of allo-centric priors, which might underlie hallucinations, delusions of control but also, under certain circumstances, the enhancement of “judgments of agency.” We discuss the consequences of such a model, and potential courses of action that could lead to its falsification.

## Introduction

In this article we will outline a computational account of perception and its disruption in psychosis. We will focus on the impact that actions have on the dynamics of perception. We will pay particular attention to how those dynamics may serve as grounds to infer agency over outcomes, and ownership of the body mediating the actions. Both ownership and agency are perturbed in people with psychotic illnesses like schizophrenia. Such perturbations manifest as profound departures from the consensual sense of how bodies work, how intentions become manifest and how agency is ascribed. For example, someone with psychosis may believe that another agent is controlling their thoughts or actions against their will (passivity phenomena) and they may perceive agents alien to themselves talking inside their head [auditory verbal hallucinations (AVH)].

The framework we develop is grounded in notions of Bayesian inference and belief updating. Put simply, perceptions (of the self, the world, and their interaction) are inferences to the best explanation (abductions) of what would need to be the case in order for the data (from the world, body, and brain) to make sense. Those inferences are based on a model of what typically happens, combined with new data. We will argue that these inferences across sources of information (external world and internal milieu) are weighted by the reliability of those sources, if one stream becomes noisier, the others are given priority, and, given priority, beliefs about those sources can be self-reinforced and become rigidly immune to updating in light of new circumstances, just as we observe in the clinic from people with psychotic illnesses.

## Robots and Predictions

We begin with a brief historical tour of the development of computational ideas relevant to action, perception, agency, and their disturbance in psychosis. Artificial intelligence—the construction and programming of intelligent machines, in cognitive science for the purpose of model building, theory construction, and hypothesis testing—has long been linked with psychiatry. In 1966, the computer scientist Joseph Weizenbaum created an early chatbot that searched for keywords in conversations conducted with human typers; if the human used one of those words, the program would use it in its reply. If not, it would offer a generic response. It was meant to mimic a psychotherapist (Weizenbaum, 1976). He named it ELIZA. In 1972, Kenneth Colby, then at Stanford created another program, PARRY—a bot that tried to model the behavior of a person with paranoia. That is, PARRY was constructed to behave as though espousing false beliefs of being harassed, subjugated, and persecuted, accused, mistreated, wronged, tormented, disparaged, vilified, and so on, by malevolent others, either specific individuals or groups. At the time, psychodynamic theories of paranoia prevailed—people were paranoid in order to protect themselves from the distress of shame and humiliation. Blaming others—the theory went—repudiated one's belief that they were to blame for an inadequacy. Parry has an interpretation module and an action module, and, through cycles of interaction with an interviewer, he progressively increments the weighting on beliefs that the interviewer has a poor opinion of him (Colby et al., 1971). Eliza and Parry interacted from different coasts of the US, via the nascent internet, and the results were amusing (Garber, 2014). Whilst they appeared to espouse knowledge and beliefs, these agents were really interacting via stimulus-response rules. They have only a shallow concept of “self,” and many of the apparently paranoid behaviors that Parry evinced were hard coded based on actual patient responses. Parry and Eliza were far from having world knowledge, let alone knowledge of themselves as agents whose communicative acts impacted others.

More recently, the late Ralph Hoffman, who pioneered computational psychiatry, built and experimented with computational patients, network-based models of verbal cognition tasked with remembering brief narratives (Hoffman et al., 2011). Central to this function in the network, as in neural network models, is prediction error, the mismatch between input and retrieval. When model prediction errors were artificially elevated, the model misremembered narratives, inserting itself into stories. A perturbation of narrative agency. This approach was formally embodied by Yamashita and Tani (2012) who inserted a predictive coding architecture into a humanoid robot; with arms and a head [(Yamashita and Tani, 2012); see also Ohata and Tani, 2020, for a similar account of multimodal, imitative interaction of agents]. It had proprioceptive inputs from its arm joints and visual inputs that were modulated by the position vectors of its neck joints. The robot was confronted with a goal-object to be manipulated. Its task was to pick up and put down the object if it is in one position, and not if the object is in another position. Sometimes the experimenter would move the object between positions. The recurrent neural network that learned and executed the task was hierarchical and imbued with top-down predictions (intentions) and perceptual inputs. Mismatches between the intended and experienced events—prediction errors—were used to learn the task contingencies. If errant prediction errors were introduced to the network, the robot began to behave erratically, switching actions and perseverating- much like people with psychosis when making decisions under uncertainty. More recently, the same authors found that aberrant prediction errors can induce excessively strong priors in the same preparation (Idei et al., 2018). It is intriguing how, despite the mercurial increase in AI research, robotics has not tended to follow (Dennett, 1994). We posit that Tani et al. work does speak to embodiment, but perhaps not to the sense of conscious agency. We do not claim that a body is required for consciousness, since people with tetraplegia retain conscious experiences. They do however, experience agency differently, interacting with the world through effectors over which they retain some agency, like their eyes or mouths. This leads to an experience of dissociation and a much denuded sense of agency (Leggenhager et al., 2012). In psychosis, the agency change is different, it is a sense of too-little agency for some events (thoughts and actions) and too-much agency for others (outcomes, external events) (Moore and Fletcher, 2012). A kernel of the present paper is how strong priors and aberrant prediction errors can co-exist in the same brain and how those computational departures give rise to perturbed sense of agency over thoughts and actions and ultimately, hallucinations and delusions.

## Conflicting Accounts

One influential theory of psychotic symptoms, hallucinations and delusions, posits that they are verbal thoughts, subvocal speech (in the case of hallucinations) or movements (in the case of passivity delusions) that are misattributed to an outside source (another agent that is communicating or controlling) (Jones and Fernyhough, 2007). This arises from compromised efference copy signals—“copies” of motor signals that are sent to sensory processing regions, rather than being sent to effectors, depositing a prediction of the expected sensory consequences of the action. Such self-induced stimulation is attenuated and may also underwrite agency attribution: I infer that I am the author of actions that proceed as expected, however, sufficient deviation from the predicted sensory consequences of actions invites the inference that another agent was involved. For hallucinations, there is some evidence for impaired efference copies of speech relating to hallucination severity, although by no means consistently. For passivity delusions, there is evidence for a failure in predictive motor cancellation that correlates with both hallucinations and delusions, in the realm of eye-movements and force-matching. If one conceives of efference copies as a kind of prior, these would be evidence for weak priors in people with psychosis that are related (perhaps) to the genesis of symptoms.

There is an alternative, based on the phenomenology of these symptoms. In particular their imperviousness to intersubjective data. That is, hallucinations and delusions do not respond well to the corrective influences of others. They are sustained despite overwhelming contradictory evidence. One might conceive of them not as relating to weak priors, but rather strong priors. If perception is an inferential process (Von Helmholtz, 1878), that inference that is optimized by prior knowledge about probable candidates (Von Helmholtz, 1866). The weighting of priors and current data is achieved by comparing their relative precision or inverse variance. If we are more confident in the data, they override our priors, if priors are more precise than sensory inputs, they will dominate inference and prediction errors will be ignored (Friston and Stephan, 2007; Friston and Kiebel, 2009; Feldman and Friston, 2010; Teufel et al., 2013). Hallucinations might arise when prior predictions exert an inordinate influence over perceptual inferences, creating percepts with no corresponding stimuli at all (Friston, 2005; Powers et al., 2016).

Indeed, in healthy volunteers who have undergone a training period that establishes an association between two stimuli, perceptual experiences of one stimulus (i.e., a tone) can occur in the absence of sensory input, conditional on the presentation of another stimulus (i.e., a visual stimulus) (Seashore, 1895), akin to a conditioned reflex (Pavlov, 1928; Ellson, 1941). More recently, visual-auditory conditioning has been employed to demonstrate that voice-hearing patients are significantly more susceptible to this effect than patients without hallucinations and controls (Kot and Serper, 2002). We recently showed that this effect is mediated by strong prior beliefs, that those priors are stronger in people who hallucinate, and that people with a diagnosed psychotic illness are less likely to update those prior beliefs in light of new evidence (Powers et al., 2017). Critically, the neural circuit underlying these conditioned phenomena—including superior temporal gyrus and insula—largely overlapped with the circuit engaged when patients report hearing voices in the scanner (Jardri et al., 2011; Powers et al., 2017). These studies underline the role of learning and, more specifically, a bias toward learned top-down information in the genesis of AVHs. Other studies, that probed the effect of high-level priors on bistable visual perception, came to similar conclusions (Schmack et al., 2013, 2017). Further support for this so-called strong prior account of hallucinations comes from findings that prior knowledge of a visual scene confers an advantage in recognizing a degraded version of that image (Teufel et al., 2015) and that patients at risk for psychosis—and, by extension, voice-hearing—were particularly susceptible to this advantage, and its magnitude correlated with hallucination-like percepts. Similarly, there is a version of this effect in audition; voice-hearing participants appear to have an enhanced prior for speech in degraded auditory stimuli even when not explicitly instructed (Alderson-Day et al., 2017). That is, speech is perhaps the most salient biological signal for our species, the auditory system of hallucination prone individuals may be pre-disposed to inferring speech. Likewise, the feeling of a lack of agency for our actions coupled with the experience that we are moving demands an explanation. All actions have a cause (internal or external) and agency typically accompanies self-generated movements. When agency is absent (i.e., the self is not the cause of the action), who or what might be causing that movement?

## The Sense of Agency

We constantly act to change our environment. Some actions are self-initiated, driven by our intentions and our expectations, while others are driven by external forces. For most of us, the distinction between voluntary and involuntary actions happens automatically, and is intimately related to the presence (or not) of a sense of agency. We define the sense of agency (SoA) as the experience of being in control of one's own actions and, through them, of events in the external world (Gallagher, 2000; Haggard, 2017). It constitutes, together with the sense of ownership [the experience that “my body” belongs to me (Tsakiris, 2017)], a key feature of self-consciousness (Braun et al., 2018) and underpins important concepts that define the human condition, such as free will and criminal responsibility (Haggard, 2017).

Despite its apparent unity, SoA consists of several components. An important distinction needs to be drawn between a “*feeling of agency”* (FoA) and a “*judgment of agency”* (JoA) (Synofzik et al., 2008a; Moore, 2016). The former can be experienced pre-reflectively and represents the non-conceptual feeling of control that colors our voluntary actions. On the contrary, JoA corresponds to a higher-level, conceptual construct that can be defined as “the ability to refer to oneself as the author of one's actions” (De Vignemont and Fourneret, 2004). The two levels of agency depend on each other [for example, FoA is a strong cue suggesting authorship of an action; “FoA is necessary but not sufficient for JoA” (Haggard and Tsakiris, 2009)] but, as recent studies have shown, they remain largely dissociable (Ebert and Wegner, 2010; Strother et al., 2010; Dewey and Knoblich, 2014; Borhani et al., 2017).

Several implicit and explicit measures have been used to measure SoA, probing its different components. Implicit measures are considered as more appropriate for quantifying FoA, since they approach agency indirectly and avoid conscious judgments. One of the first implicit measures that was employed is *sensory attenuation*: the perceived intensity of sensations resulting from voluntary actions is diminished compared to sensations caused by involuntary (or external) actions [e.g., we cannot tickle ourselves (Blakemore et al., 2000b)]. Another implicit measure, considered as the hallmark of volition, is *intentional binding*: actions and the ensued outcomes are perceived closer together when the action is voluntary, resulting in a subjective contraction of time (Moore and Obhi, 2012). Indeed, in a series of studies Haggard et al. found that intentional binding occurred only in the case of voluntary actions, while involuntary actions evoked by Transcranial Magnetic Stimulation (TMS) of the motor cortex had the opposite result [repulsion (Haggard et al., 2002; Haggard and Clark, 2003)]. Since then, scientists have discovered links between binding and predictability (Moore and Haggard, 2008; Wolpe et al., 2013), associative learning [binding is enhanced by surprise (Moore et al., 2011a)], instrumental control (Borhani et al., 2017) and the fluency of action selection (Chambon et al., 2014).

On the other hand, explicit measures directly ask participants to make judgments about their agentic experience (Moore, 2016). In one example, participants are asked to make a hand movement and then see the same movement or a similar movement performed by another hand on a screen. In some cases and unbeknownst to the participant, a spatial or temporal distortion is added to the visual feedback. When asked whose hand they see on the screen, many participants misperceive the other hand as their own (especially in cases of no or small distortions), indicating the existence of a self-attribution bias (Farrer et al., 2003; Tsakiris et al., 2005; Hauser et al., 2011a). Other researchers asked the participants to make judgements about the feeling itself (Sidarus et al., 2013; Chambon et al., 2015). They found that parameters such as compatibility of priming, predictability and action-outcome delay profoundly affected participants' responses.

Disturbances of SoA are a common feature of psychotic disorders, such as schizophrenia (for a summary of the main empirical findings, see Table 1). Patients feel having no control over their actions and thoughts, which are instead controlled by external agents [passivity symptoms (Waters and Badcock, 2010)]. The presence of those passivity symptoms speaks to a diminished SoA in schizophrenia patients. However, carefully designed experiments found enhanced intentional binding (Haggard et al., 2003; Voss et al., 2010) and a stronger self-attribution bias in patients with schizophrenia (and passivity symptoms in particular) (Daprati et al., 1997; Franck et al., 2001), implying an exaggerated self-consciousness rather than a diminished sense of self (Hur et al., 2014). The apparent paradox is still not fully resolved, but evidence suggests a two-level impairment, namely an impairment in predictive components of agency (components related to processing occurring prior to action initiation (e.g., motor predictions, fluency of action selection etc.); possibly related to passivity symptoms), followed by an enhancement of retrospective processing (it includes processing that takes place after the action has been completed and feedback has been received; perhaps resulting in over-attribution) (Synofzik et al., 2010; Voss et al., 2010).

**Table 1:** Sense of agency/ownership (implicit and explicit measures) in psychosis: main empirical findings.

**References**	**Population (sample size)**	**Paradigm**	**Main findings**
Malenka et al. (1982)	SCZ (14)	Tracking task (Error corrections)	SCZ: Fewer error corrections without external (visual) cues
Frith and Done (1989)	SCZ + AP (23) (P+: 10; P–: 13)	Motor task (Error corrections)	SCZ: Fewer error corrections without external (visual) cues
Daprati et al. (1997)	SCZ (30) (H+: 13; DC+: 7; H+DC+: 6; H–DC–: 10)	Recognition task (“Is that my hand on the screen?”)	H+, DC+: More false self-attributions
Blakemore et al. (2000a)	SCZ (23) + AD (18) (H+: 17; H+P+: 6; H–P–: 24)	Sensory attenuation task (tactile stimulation)	H+, P+: No sensory attenuation of self-produced tactile sensations
Franck et al. (2001)	SCZ (24) (P+: 6; P–: 18)	Recognition task (“Is that my hand on the screen?”)	P+: More false self-attributions
Delevoye-Turrell et al. (2002)	SCZ (16) (DC+: 6; DC–: 10)	Force adjustment task	DC+, DC–: No improvement of efficiency of motor response in self- vs. externally- imposed condition
Haggard et al. (2003)	SCZ (8)	Intentional binding task	SCZ: Stronger binding between actions and outcomes
Allen et al. (2004)	SCZ (28) (H+D+: 15; H–D–: 13)	Recognition task (“Is this my voice?”)	H+D+: More misidentifications of their own speech as alien—correlation with severity of hallucinations
Knoblich et al. (2004)	SCZ (27)	Motor task (implicit—explicit error corrections)	SCZ (with symptoms): Impaired explicit detection of action-outcome mismatches/intact implicit corrections
Lindner et al. (2005)	SZC (14)	Sensory attenuation task (Smooth-pursuit eye-movement task)	SCZ: Less sensory attenuation (stronger reafference) —correlation with severity of delusions of control
Shergill et al. (2005)	SCZ (19)	Sensory attenuation task (Force-matching task)	SCZ: Less sensory attenuation (less underestimation of self-generated force)
Synofzik et al. (2010)	SCZ (20)	Task 1: Detection of discrepancies between action (pointing) and distorted visual feedback Task 2: Estimation of direction of pointing with or without distorted visual feedback	SCZ: Task 1—Higher thresholds for detecting action-outcome discrepancies; Task 2—More adaptation of estimates to feedback; More variable estimates; Variability of estimates (in the absence of feedback) correlated with delusions of control and detection thresholds from task 1
Teufel et al. (2010)	CTR (30)	Sensory attenuation task (Force-matching task)	Participants with higher delusion-proneness (PDI score) exhibited weaker sensory attenuation
Voss et al. (2010)	SCZ (24)	Intentional binding task	SCZ: Impaired predictive component of action awareness (weaker effect of outcome predictability—correlated with positive symptoms) —enhanced retrospective component (presence of the outcome)
Hauser et al. (2011a)	SCZ (30); PP (30)	Recognition task (“Did I produce this tone?”)	Both SCZ and PP: More false self-attributions—self-attribution bias correlated with passivity symptoms
Hauser et al. (2011b)	PP (30)	Intentional binding task	PP: Stronger intentional binding—both predictive and retrospective influences were stronger—predictive influences correlated with ego-psychopathology (IPP score)
Moore et al. (2011b)	CTR_Ket (14)	Intentional binding task	Ketamine enhances binding—correlation with aberrant bodily experiences
Thakkar et al. (2011)	SCZ (24)	Rubber Hand Illusion (RHI)	SCZ: Stronger RHI (both implicitly and explicitly measured)—self-reported strength of RHI correlated with schizotypy in CTR
Maeda et al. (2012)	SCZ (30)	Agency attribution task	SCZ: Excessive sense of agency (even when outcomes precede actions)
Renes et al. (2013)	SCZ (23)	Agency attribution task (explicit condition: intentions/implicit condition: priming)	SCZ: Enhanced self-agency in explicit condition (not different from CTR)—Less enhancement than CTR in implicit condition
Hur et al. (2014)	Meta-analysis−25 studies SCZ (690)		Self-disturbance in SCZ: distortions in body-ownership, self of agency (enhanced) and self-reported subjective experiences
Moore and Pope (2014)	CTR (35)	Agency attribution task with video stimuli	Presence of intentionality bias. The bias is stronger in individuals with stronger schizotypal traits
Koreki et al. (2015)	SCZ (30)	Agency attribution task	SCZ: Excessive sense of agency (even for action-outcome delays longer than 1s)
Garbarini et al. (2016)	SCZ (20)	Bimanual coupling task (bimanual condition: participants draw lines with one hand and circles with the other—modified condition: participant draws lines with one hand while observing examiner drawing circles)	SCZ: Same interference effects in bimanual condition—stronger interference in modified condition
Lemaitre et al. (2016)	CTR (ST+: 27; ST–: 27)	Sensory attenuation task (tactile stimulation)	Self-applied tactile stimulations are felt to be more ticklish by healthy individuals high in schizotypal traits—self-tickling was associated with passivity experiences
Voss et al. (2017)	SCZ (14)	Agency attribution task + priming	SCZ: Similar effects of priming on motor performance—no effect of priming on sense of agency (contrary to CTR)
Whitford et al. (2017)	CTR (110)	Sensory attenuation task (tactile stimulation)	Participants with stronger schizotypal traits (SPQ score) exhibited weaker sensory attenuation
Graham-Schmidt et al. (2018)	SCZ (51) (Current P+: 20; Past P+: 10; P–: 21)	Projected Hand Illusion (PHI)	P+ (current or past): Less difference in agency between active and passive movements when assessing agency over their own hand

*SCZ, Schizophrenia patients; AP, Affective Psychosis; AD, Affective disorder; PP, Prodromal Patients; CTR, Controls; CTR_Ket, Controls given Ketamine; H+, With hallucinations; H–, Without hallucinations; D+, With delusions; D–, Without delusions; DC+, With delusions of control; DC–, Without delusions of control; P+, With passivity symptoms; P–, Without passivity symptoms; FTD+, With formal thought disorder; ST+, High schizotypal traits; ST–, Low schizotypal traits*.

The computational underpinnings of agency have also been lively debated over the past 30 years. According to the influential *comparator model* (Feinberg, 1978; Blakemore et al., 2000a; Blakemore and Frith, 2003), SoA relies on the motor system that is responsible for initiating and controlling self-generated movements, based on the principles of optimal control theory (Wolpert et al., 1995; Wolpert and Ghahramani, 2000). More particularly, the brain predicts the sensory consequences of self-initiated actions through the use of *forward models* (Wolpert and Kawato, 1998). A copy of the motor prediction [*corollary discharge*; often called *efference copy* (Feinberg, 1978)] is sent to the sensory areas, suppressing predictable inputs (proprioceptive but also visual, auditory etc.). This sensory attenuation of self-generated inputs (discussed above) ultimately gives rise to the feeling that one is in control of their own actions.

Despite its success, several criticisms against the comparator model have been raised, largely based on its inability to account for JoA (e.g., Synofzik et al., 2008a). According to the theory of *apparent mental causation*, put forward by Wegner and Wheatley, SoA does not rely on the motor signals that initiated the action, but on generic inferential processes (Wegner and Wheatley, 1999). In a nutshell, this theory suggests that (1) if an action is preceded by an intention, (2) if the action is compatible with that intention and (3). if the intention is the most likely cause of the action, then the action is attributed to one's self. Intriguingly, this theory is not based on “private” mechanisms (such as the motor signals) and thus, it can be generalized to other peoples' actions. More recently, several theorists tried to combine the above models, bridging the gap between motor and inferential processes and, more generally, between FoA and JoA (Synofzik et al., 2008a,b; Moore and Fletcher, 2012; Moutoussis et al., 2014; Kahl and Kopp, 2018; Legaspi and Toyoizumi, 2019).

## Reconciling Conflicting Accounts

In the previous sections we argued that two different riddles have been puzzling researchers for decades. Namely:

- Are hallucinations and delusions due to strong or weak priors [i.e., strong priors (Sterzer et al., 2018; Corlett et al., 2019) vs. weak corollary discharge (Blakemore et al., 2002; Thakkar et al., 2017) and a loss of agency for one's inner speech (Jones and Fernyhough, 2007)]?
- Relatedly, do schizophrenia patients have an exaggerated or a diminished SoA?

Paradoxically, in both cases there is evidence supporting strong and weak priors, weak corollary discharge, misattributed inner speech, exaggerated, and diminished agency (though typically not at the same time in the same people with psychosis).

In this section we advance a conceptual model which, we believe, can reconcile those (seemingly) contradictory accounts. We argue that there is more than one generative model in the brain, and that ego- and allo-centric models operate in tandem. In brief, there are inferences (related to actions) that need to represent and account for the impact of self on perception (ego-centric) and there are inferences that do not need such accounting (allo-centric). There may be a precision-weighted trade-off between which source is drawn upon for inference, especially in the case of agency attribution. Such a trade-off would allow for aberrant corollary discharges and strong priors in the same individual, both of which contribute to symptom genesis. Additionally, by postulating that each one of the two hierarchies is responsible for a different level of agency attribution, our model can predict both exaggerated and diminished SoA, depending on the experimental context. We note that a detailed mathematical description is beyond the scope of this paper and will be presented in future publications.

### The Model

**Figure 1:**
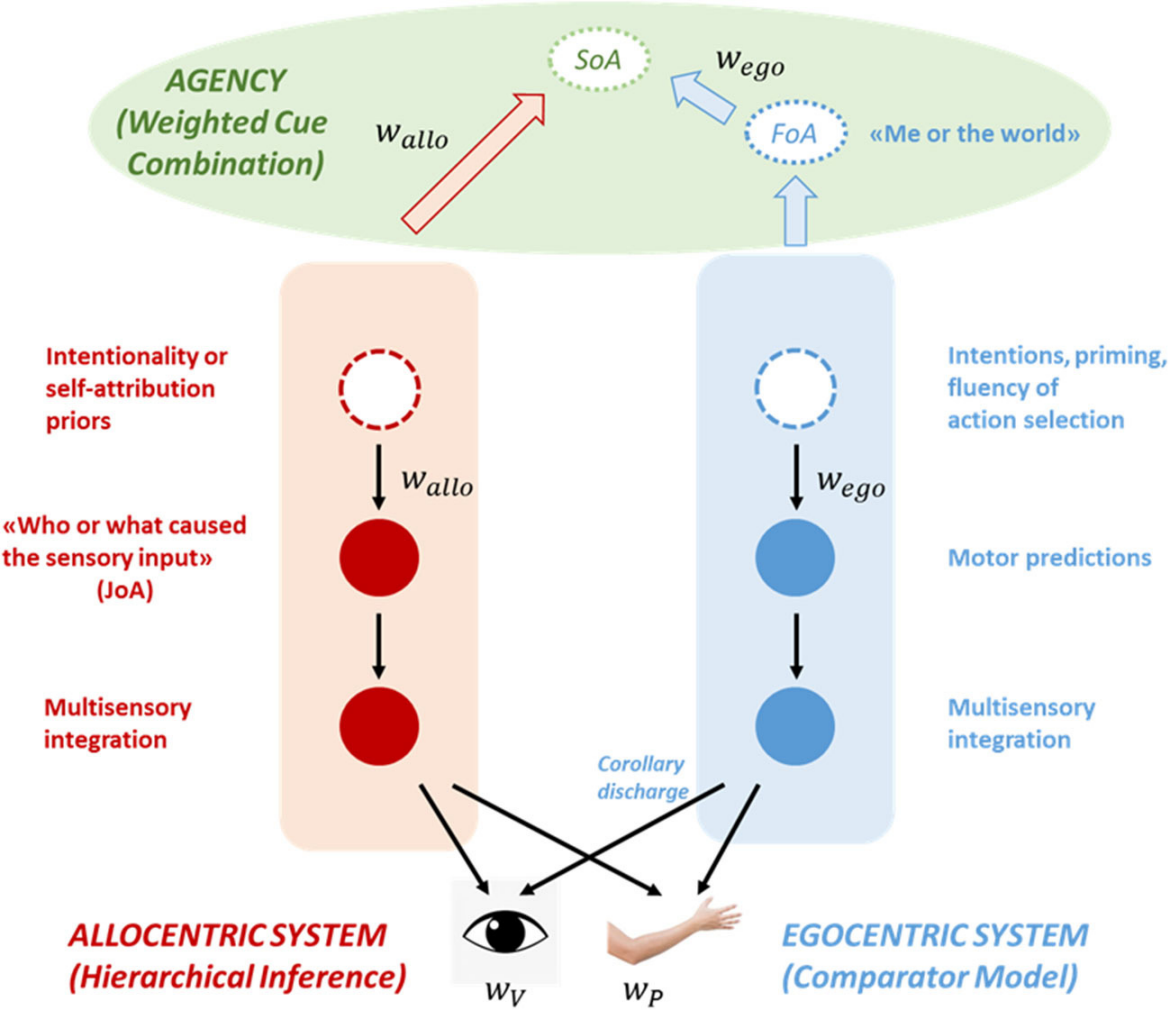
An illustration of the model. The model consists of two hierarchies, an ego-centric and an allo-centric system, that operate in tandem and interact at the sensory level. The ego-centric system (in blue) is part of a sensorimotor loop and implements a comparator model. A copy of the motor command (transformed via a forward model into a motor prediction about the sensory outcomes of the action) is sent to the sensory areas where it suppresses self-generated (i.e., predictable) inputs. Motor predictions can be modulated by higher-level factors such as intentions or the fluency of action selection. The allo-centric system (in red) represents generative causal models of the world, including the self among the potential causes. According to predictive coding, allo-centric predictions (like motor predictions) explain away predictable inputs, but unlike the ego-centric system those inputs are not necessarily self-generated. Allo-centric predictions are also modulated by higher-level priors such as an intentionality or a self-attribution bias. Both types of predictions and the sensory inputs are weighted according to their reliability (*w*_*ego*_, *w*_*allo*_, and *w*_*V*_, *w*_*P*_, respectively). Crucially, both systems make inferences about different levels of agency. The ego-centric system implements a private mechanism that makes a self-world distinction and gives rise to a feeling of agency (FoA) when motor predictions and inputs are in good match. The allo-centric system on the other hand generates judgments of agency (JoA) based on generic inferential mechanisms, by comparing multiple hypothesis about the cause of a certain outcome (“Me” vs. “External agent” vs. “External non-agentic cause” vs…). The different components of agency are then fed-forward to an agency-attribution system (in green), where they are combined according to a weighted cue combination mechanism that gives rise to a higher-level sense of agency (SoA).

Our model is illustrated in Figure 1. It consists of two hierarchies operating in parallel: An ego-centric hierarchy, predicting self-generated inputs, and an allo-centric hierarchy, implementing more general inferences regarding the state of the world. Interestingly, the 2 hierarchical systems are related to each other, as they receive bottom-up information from the same sensory systems [e.g., retina and primary visual cortex in the case of visual inputs; proprioceptors and cerebellum in the case of proprioception (Shergill et al., 2014)].

The ego-centric system is part of a sensorimotor loop, that controls and optimizes the trajectories of movements (Wolpert and Ghahramani, 2000). Copies of the motor commands (i.e., efference copies) are transformed into motor predictions about the sensory consequences of self-generated actions through the use of internal predictors, the forward models (Wolpert and Kawato, 1998). Those motor predictions (i.e., corollary discharges) are then weighted according to their reliability (*w*_*ego*_) and sent to sensory areas, where they attenuate precision-weighted (*w*_*V*_, *w*_*P*_ etc.) self-generated inputs (primarily proprioceptive but also visual, auditory etc.; Wolpert et al., 1995; Blakemore and Frith, 2003; Körding and Wolpert, 2004). Importantly, the motor predictions and their precision can be modulated by various factors such as intentions or cues preceding action initiation (priming effects or fluency of action selection). For example, fluent selection of the appropriate action might have profound effects on the strength of the efferent signals (Chambon et al., 2014).

The allo-centric system on the other hand implements more generic predictive processing based on the principles of hierarchical Bayesian inference. Very briefly, that means that the allo-centric system learns and represents causal models of the world and inverts those models to estimate the most probable cause of the sensory input [self-produced or not (Von Helmholtz, 1866; Clark, 2013)]. According to predictive coding theory, high- level predictions (weighted according to their reliability *w*_*allo*_) explain away sensory inputs (*w*_*V*_,*w*_*P*_ etc.), in the same way motor predictions suppress self-generated inputs (Friston and Kiebel, 2009). When there is a mismatch between predictions and inputs, a prediction error signal is generated which updates the current model. Importantly, this constructive view of perception implies that percepts are not pure representations of sensory inputs, instead they are biased by prior knowledge, which might be learnt through experience [e.g., empirical priors (Friston, 2009)] or hard-coded through evolution [e.g., “light comes from above” (Mamassian and Landy, 1998; Dobbins and Grossmann, 2010)]. It's worth noting that learning can be driven both by the reliability of the cues and by uncertainty (Corlett, 2020).

Crucially, both systems contain the necessary machinery to make inferences about the contribution of the self in the generation of the inputs and thus, about agency (Wegner and Wheatley, 1999; Blakemore and Frith, 2003). The ego-centric system is an implementation of Frith's comparator model (Blakemore et al., 2000b). More particularly, ego-centric (motor) predictions suppress sensory inputs only in case they are predictable, that is, if they are self-generated. Consequently, sensory attenuation should be followed by a feeling that one is control of their own actions, in other words, they should experience a FoA. We should highlight here that this is a “private” mechanism, only applicable to one's self (Synofzik et al., 2008a; Carruthers, 2009). That means that the ego-centric system does not have the necessary mechanisms to attribute agency to someone else; it can only decide between “me” and “the world.”

The allo-centric system relies on more eight general inferential mechanisms, therefore it can choose between different internal and external causes (“me,” “you,” an object etc.), potentially underwriting judgments of agency (JoA). Those inferences can rely on sensory inputs (e.g., movement of a hand, moving lips etc.) but also on priors regarding the intentions of others. Furthermore, those agency-related inferences might also be driven by hardwired biases such as the intentionality bias (Rosset, 2008; Sidarus et al., 2013) or a self-attribution bias (Farrer et al., 2003; Tsakiris et al., 2005; Hauser et al., 2011a). In the context of Bayesian inference, those biases can be conceptualized as additional priors or hyperpriors (priors on hyperparameters that control the shape of the prior distributions). Although those additional priors can reduce the accuracy of the agency attribution mechanism, they might enhance social bonding, underpin “theory of mind” or increase self-esteem (see also Garety and Freeman, 1999; Schwarz et al., 2016).

Following previous theoretical suggestions (Synofzik et al., 2008a; Moore and Fletcher, 2012; Kahl and Kopp, 2018), we postulate that FoA and JoA are combined to generate a higher-level SoA via a precision-weighted cue combination mechanism, where the 2 weights can be related to the precision of the ego-centric and allo-centric predictions, respectively. For example, a partial attenuation of the input by the ego-centric predictions might result in a lack of a FoA, which can in turn override the allo-centric intentionality priors (and a potentially positive JoA), resulting in the belief that we are not the author of the action.

In the next section, we describe the implications of the model in the case of schizophrenia. In particular, we suggest that the interactions between and within the two hierarchies of inference can reconcile the apparent contradictions (Sterzer et al., 2018; Corlett et al., 2019).

### Schizophrenia^1^: From Weak Motor Predictions to Strong Allo-Centric Predictions

**Figure 2:**
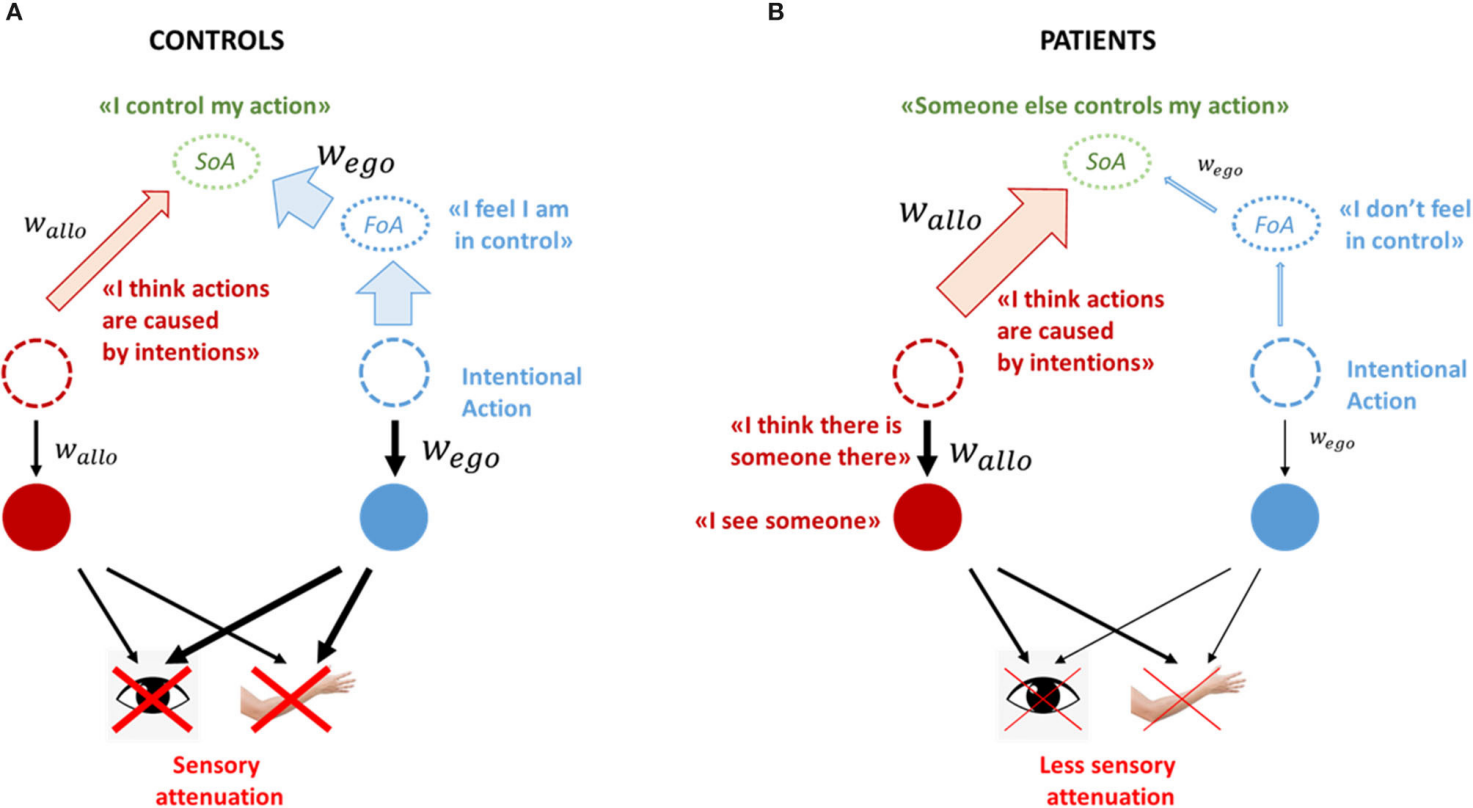
Healthy controls vs. Schizophrenia patients. **(A)** In a well-functioning system, predictions (both ego-centric and allo-centric) are weighted according to their reliability. When an intentional action is initiated, strong corollary discharge signals explain-away self-generated inputs, which in turn give rise to a FoA and a sense that one is in control of their actions. Within the allo-centric system, that results in optimal perceptual inferences **(B)** When motor predictions are under-weighted (e.g., in schizophrenia), self-generated inputs cannot be explained away, resulting in a feeling that one is not in control of their actions. The unsuppressed inputs flood into the allo-centric system, which is overflowed with noisy and inherently unpredictable information. To compensate for that, it increases the weight of high level allo-centric priors, including agency-related priors such as the intentionality bias. Strong priors have an effect both on perceptual decision making and on agency-attribution: first, percepts are mainly driven by priors, rendering the system susceptible to hallucinations. Second, the enhanced intentionality bias, combined with the lack of a FoA, bring about the false belief that an external agent is in control of one's own actions, i.e., a delusion of control.

There is an abundance of evidence that interactions between motor and perceptual systems are crucial for both functions (Faivre et al., 2015). A well-functioning perceptual system (i.e., a system that attributes precise weights to priors and sensory inputs, according to their reliability) makes accurate perceptual decisions, which in turn can lead to meticulous adjustments of the self-generated movements, through the operation of sensorimotor loops. Vice versa, intact corollary discharges explain away the unnecessary self-induced sensory signals, preventing them from affecting allo-centric inferences (Figure 2A). An interesting example of this fine-tuned interaction is saccadic suppression and the ensued visual stability during eye-movements (Melcher, 2011): although we make several saccades every second, whose peak speed can reach several hundreds of degrees/sec, we perceive no changes in our visual field, an effect that is usually attributed to efferent inhibitory motor signals (corollary discharge) (Cavanaugh et al., 2016). Importantly, the optimal integration of the allo- and ego- centric predictions also results in precise agency-estimates, based on the accurate calculation and combination of the FoA and JoA.

What happens if we selectively impair corollary discharge signals, as described in schizophrenia (Blakemore et al., 2002; Synofzik et al., 2010; Thakkar et al., 2017)? Motor predictions cannot explain away self-generated signals, resulting in a reduced sensory attenuation of those sensations (Figure 2B; Blakemore et al., 2000a; Shergill et al., 2005) and a diminished FoA. That explains why patients with schizophrenia do not feel in control of their own actions, however it does not explain why they attribute their actions to an external agent (Frith, 2005).

Ego-centric and allo-centric hierarchies work in tandem. We argue that impairments in one system (e.g., weak corollary discharge) have a profound effect in the opposite system as well (Corlett et al., 2019; Thakkar and Rolfs, 2019). In the case of schizophrenia patients, the un-attenuated self-generated sensory signals would penetrate in the allo-centric hierarchy, flooding it with noisy, inherently unpredictable information (e.g., rapidly changing visual inputs during saccadic movements; see also Seal et al., 2004; Jones and Fernyhough, 2007; Alderson-Day and Fernyhough, 2015) and also resulting in low level perceptual abnormalities (e.g., blurred images, changes in perception of size or color etc.). Various experimental findings corroborate this idea: first, patients exhibit deficient saccadic suppression, which results in unstable visual images during movement (pseudo-movements) (Krekelberg, 2010; Thakkar and Rolfs, 2019); second, self-generated, subvocal speech, picked by throat microphones, has been causally associated with certain types of AVH (Gould, 1950; Bick and Kinsbourne, 1987), suggesting that self-generated stimuli receive special attention and are mis-processed by patients; third, when people report AVH in the scanner, their speech network (including both speech production and reception areas) is engaged (Jardri et al., 2011). This penetration gives rise to strong prediction error signals, which are propagated toward higher levels, constantly updating the internal models. Additionally, given the tight connection between saccades and spatial attention, impaired corollary discharge signals might also give rise to attentional problems, including the aberrant salience attributed to random stimuli in the environment (Thakkar and Rolfs, 2019). In both cases, the world would seem unstable, unpredictable and strange. We suggest that the allo-centric system compensates for the overwhelming bottom-up signals by increasing the precision of high-level allo-centric priors (Adams et al., 2013; Schmack et al., 2013, 2017; Powers et al., 2017; Sterzer et al., 2018; Corlett et al., 2019). This compensatory mechanism would alleviate the strong impact of the self-generated signals by increasing the relative contribution of the priors in allo-centric inferences, resulting in more stable and less chaotic percepts. Despite its beneficial effect, this overreliance on priors also renders the system more vulnerable to hallucinations (Figure 2B). Indeed, auditory hallucinations are one of the most prominent symptoms in schizophrenia and have been repeatedly associated with strong priors (Teufel et al., 2015; Powers et al., 2016, 2017). Can strong priors also explain the content of hallucinations and delusions (e.g., predominantly negative content of AVH, technical delusions etc.)? This is not an unreasonable speculation, especially if also take into account the affective and cultural forces that “shape” those priors (Škodlar et al., 2008; Laroi et al., 2014).

This enhancement of allo-centric priors also has significant effects on the SoA. Combined with the down-regulation of the motor predictions, it means that the JoA gains a particular significance, compared to FoA. But JoA is subject to various biases, including an intentionality bias (Rosset, 2008). This means that individuals that overweight priors would have a stronger tendency to attribute actions to hidden intentions, thus perceive volitional behaviors even when there are none. Taken together, they explain the phenomenology of delusions of control, where people do not feel in control of their own actions and attribute them to external forces (Frith, 2005).

Interestingly, the same impairments can also explain the opposite pattern, notably the tendency of schizophrenia patients with passivity symptoms to over-attribute certain actions to themselves in recognition tasks (Daprati et al., 1997; Franck et al., 2001). The key observation here is that in those tasks ego-centric predictions are largely irrelevant; a FoA is dissociated from the perceptual decision “is this my hand.” In this case, a SoA (and consequently the perceptual decision) depends first and foremost on allo-centric JoA. But JoA is also subject to a self-attribution bias [(Garety and Freeman, 1999); the intentionality bias is also at play], which is enhanced due to the overweighted priors. Consequently, patients can over-attribute and under-attribute actions to themselves, depending on the experimental context. Similar arguments can be put forward to explain delusions of reference (Maeda et al., 2012), while it's an open question whether similar mechanisms could explain other first-rank symptoms such as thought insertion or made feelings (Vosgerau and Newen, 2007; Frith, 2012).

In short, we described a conceptual model that reconciles contradictory accounts of schizophrenia, namely whether patients over-weight or under-weight their priors, and whether they have an exaggerated or a diminished SoA. The model can explain various state symptoms (symptoms that manifest themselves during full-blown psychotic episodes, such as hallucinations, delusions of control or even low-level perceptual abnormalities), it remains unclear though whether similar mechanisms could also explain trait symptoms [more permanent features of schizophrenia, also found in first-degree relatives and high-risk populations (Adams et al., 2013)] and, more importantly, different phases of the disorder, such as the prodromal phase. In the next section we describe some further predictions of the model.

## Explanatory Power and Novel Predictions

The combined impaired-corollary discharge and strong-priors account that we outlined above makes some additional predictions, some of them novel, meaning that it is a highly falsifiable theory. That said, given the conceptual nature of the described model, our predictions should be made with caution.

First, it is compatible with data suggesting both compromised motor predictions (Lindner et al., 2005; Synofzik et al., 2010; Thakkar et al., 2017) and overly strong priors (Powers et al., 2017). Importantly, because of the assumed causal link between the two, we expect an anti-correlation within the same individuals (Corlett et al., 2019); e.g., participants with less sensory attenuation and stronger re-afferent signals should also report more conditioned hallucinations. Stronger evidence in favor of our theory could be obtained from causal, virtual lesion studies such as TMS studies: stimulation of regions critically involved in ego-centric inferences such as cerebellum (Blakemore et al., 1998; Synofzik et al., 2008c) or the temporo-parietal junction (TPJ) (Hughes, 2018) should engender hallucinations in participants (Arzy et al., 2006).

More generally, our theory suggests that failures of the ego-centric system would render the perceptual system more susceptible to false percepts and hallucinations. Interestingly, recent work suggests that sensorimotor conflicts induced by a robotic system decrease the capacity to adapt confidence to task performance (metacognitive failure), increase intentional binding (potentially due to an enhanced JoA) (Faivre et al., 2020) and generate a feeling of presence (Blanke et al., 2014).

Finally, our theory makes several predictions regarding SoA and its impairments in schizophrenia and in related psychotic disorders (Hauser et al., 2011a,b; Moore et al., 2011b). Primarily, our theory predicts an anti-correlation between FoA and JoA (and the related explicit or implicit measures of FoA and JoA) within the same participants. For example, one might expect decreased sensory attenuation (an implicit measure of FoA; Shergill et al., 2005; Teufel et al., 2010) to correlate with increased self-over-attribution in recognition tasks (Daprati et al., 1997; Franck et al., 2001). Intriguingly, one might also expect judgments of ownership (JoO), whose cognitive and computational mechanisms partly overlap with those of JoA (Tsakiris, 2017), also to anti-correlate with FoA. For example, vulnerability to the rubber hand illusion [(Tsakiris and Haggard, 2005); an increased vulnerability of the RHI has been observed in schizophrenia patients (Thakkar et al., 2011)] should correlate with less sensory attenuation. Ultimately, the present theory also explains several observations about intentional binding, such as the reduced effect of priming (Voss et al., 2017) and the enhanced effect of retrospective processing (Voss et al., 2010) in schizophrenia patients, while it also predicts a decreased effect of the fluency of action selection in the same populations (Chambon et al., 2014).

## Summary and Conclusions

This paper outlines an account of inference and agency that reconciles several conflicting lines of evidence. Ego-centric and allo-centric models operate in tandem, making up the machinery required for attaining self-other distinction and thus, SoA. Ego-centric models implement corollary discharge signals that cancel out the effects of self-generated actions, subserving FoA. Allo-centric models compare several hypothesis regarding the causes of sensory inputs (including the self among the potential causes), giving rise to JoA. The different levels of agency are weighted according to their reliability and combined, ultimately forming a higher-level SoA. In schizophrenia, a failure of corollary discharges to suppress self-generated inputs results in the absence of a FoA and in a (compensatory) enhancement of allo-centric priors, which might underlie hallucinations, delusions of control but also, under certain circumstances, the enhancement of JoA.

## Data Availability Statement

The original contributions presented in the study are included in the article/supplementary material, further inquiries can be directed to the corresponding author/s.

## Author Contributions

PL and PC conceived the model and wrote the paper. All authors contributed to the article and approved the submited version.

## Conflict of Interest

The authors declare that the research was conducted in the absence of any commercial or financial relationships that could be construed as a potential conflict of interest.

## Footnotes

^1^Schizophrenia is a heterogeneous disorder, characterized by positive (psychotic), negative and cognitive symptoms. The model outlined in this paper is an effort to understand mechanistically the positive symptoms (more particularly, hallucinations and delusions of control) and not schizophrenia as a whole. Other common symptoms of schizophrenia, including negative symptoms and other types of delusions (e.g. persecutory delusions), are likely to be underwritten by different mechanisms.
